# Microwave radiation induces neuronal autophagy through miR-30a-5p/AMPKα2 signal pathway

**DOI:** 10.1042/BSR20212584

**Published:** 2022-04-19

**Authors:** Yanhui Hao, Wenchao Li, Hui Wang, Jing Zhang, Haoyu Wang, Ji Dong, Binwei Yao, Xinping Xu, Li Zhao, Ruiyun Peng

**Affiliations:** Department of Experimental Pathology, Beijing Institute of Radiation Medicine, Beijing, China

**Keywords:** AMPK (5’-AMP activated kinase), Autophagy, Microwave radiation, miR-30a-5p, Neuron

## Abstract

The potential health hazards of microwaves have attracted much more attention. Our previous study found that 2856 MHz microwave radiation damaged synaptic plasticity and activated autophagy in neurons. However, the mechanisms underlying microwave-induced autophagy were still unclear. In the present study, we established neuronal damage models by exposing rat hippocampal neurons and rat adrenal pheochromocytoma (PC12) cell-derived neuron-like cells to 30 mW/cm^2^ microwaves, which resulted in miR-30a-5p (‘miR-30a’ for short) down-regulation and autophagy activation *in vivo* and *in vitro*. Bioinformatics analysis was conducted, and *Beclin1, Prkaa2, Irs1, Pik3r2, Rras2, Ddit4, Gabarapl2* and autophagy-related gene 12 (*Atg12*) were identified as potential downstream genes of miR-30a involved in regulating autophagy. Based on our previous findings that microwave radiation could lead to abnormal energy metabolism in neurons, *Prkaa2*, encoding adenosine 5′-monophosphate-activated protein kinase (AMPK) α2 (AMPKα2, an important catalytic subunit of energy sensor AMPK), was selected for further analysis. Dual-luciferase reporter assay results showed that *Prkaa2* was a downstream gene of miR-30a. Moreover, microwave radiation increased the expression of AMPKα2 and the phosphorylation of AMPKα (Thr^172^) both *in vivo* and *in vitro*. The transfection of PC12 cells with miR-30a mimics increased miR-30a levels, reduced AMPKα2 expression, suppressed AMPKα (Thr^172^) phosphorylation, and inhibited autophagy occurrence in neuron-like cells. Importantly, miR-30a overexpression abolished microwave-activated autophagy and inhibited microwave-induced AMPKα2 up-regulation and AMPKα (Thr^172^) phosphorylation. In conclusion, microwave radiation promoted the occurrence of autophagy in neurons through the miR-30a/AMPKα2 signal pathway.

## Introduction

Microwave use is widespread in modern life. Radiofrequency, including microwaves, had been classified as 2B carcinogens in 2013. Moreover, the National Toxicology Program of U.S.A. has reported the latest evidence that microwave radiation with a frequency of 900 MHz can cause cancers such as glioma in rats [1]. The brain is considered to be sensitive to microwave radiation, as many studies have reported the damaging effects of microwave exposure on the brain [2–5]. Therefore, it is important to study the biological effects and underlying mechanisms of microwave-induced neuronal damage. Autophagy, typically referred to as macroautophagy, is a cellular process that sequesters, removes and recycles unwanted macromolecules and damaged organelles, and is of great importance in maintaining cellular homeostasis [6]. Numerous studies have shown that autophagy plays important roles in the pathophysiological processes of various diseases, including tumors and neurodegenerative disorders [7–11]. In our previous study, autophagy was found to be activated in rat hippocampal neurons following microwave exposure [12]. However, the mechanisms involved in the regulation of autophagy induced by microwave radiation remain unclear.

MicroRNAs (miRNAs) are 20–24 nucleotide noncoding RNAs that can affect protein expression at the post-transcriptional level by modulating the stability and translation of corresponding messenger RNAs (mRNAs) [13]. The roles played by miRNAs in various biological processes have received increasing attention. In our previous study, several differentially expressed miRNAs were screened from the rat hippocampus exposed to microwave radiation [14], among which miR-30a-5p (‘miR-30a’ for short) attracted our attention due to its regulatory effects on autophagy. Studies have reported that miR-30a negatively regulates Beclin1-mediated autophagy, which has emerged as a promising therapeutic target for multiple diseases, such as cerebral ischemic stroke, infection and cancers [15–19]. Additionally, we previously found that microwave radiation could induce abnormal energy metabolism in neurons [20,21]. As an energy sensor, adenosine 5′-monophosphate-activated protein kinase (AMPK) plays key roles in maintaining cellular metabolic balance and in regulating autophagy [22]. However, whether and how miR-30a and AMPK regulated microwave-mediated autophagy activation requires further elucidation.

In the present study, we showed that microwave radiation promoted autophagy occurrence and reduced miR-30a expression both in rat hippocampal neurons and rat adrenal pheochromocytoma (PC12) cell-derived neuron-like cells. Prkaa2, encoding AMPKα2, was identified as one of the downstream genes of miR-30a. Microwave radiation increased AMPKα2 expression and AMPKα (Thr^172^) phosphorylation both *in vivo* and *in vitro*. In neuron-like cells, miR-30a overexpression abolished microwave-induced AMPKα2 up-regulation and autophagy activation.

## Materials and methods

### Animals and microwave exposure

All animal experiments took place at the Beijing Institute of Radiation Medicine. All experimental procedures were performed in accordance with the National Institutes of Health Guide for the Care and Use of Laboratory Animals and were approved by the Institutional Animal Care and Use Committee of the Beijing Institute of Radiation Medicine.

Sample size was arbitrarily set to 48 (two groups with 24 animals each). Male Wistar rats (212.5 ± 7.1 g, 8-week-old, specific pathogen-free) were provided by and maintained in the Laboratory Animal Center of Beijing Institute of Radiation Medicine, where the temperature was 22 ± 2°C and the humidity 55 ± 10% on a 12-h light–dark cycle. Food and water were freely available. The rats were randomly divided into two groups: the microwave-exposed (MW) and sham-exposed (SH) groups. As described in our previous study [12], the rats in the MW group were placed in fan-shaped boxes made of plexiglass and free of metal just below the microwave source and exposed to 2856 MHz microwaves at an average power density of 30 mW/cm^2^ for 15 min, once every other day for three exposures. The specific absorption rate (SAR) was approximately 10.5 W/kg; the calculation method has been described previously [23]. The rats in the SH group were processed in parallel with those in the MW group, but with the microwave source switched off. At appointed time points, rats were anesthetized with 1% pentobarbital sodium (30 mg/kg) by intraperitoneal injection to minimize animal suffering during the procedure. The rats were then decapitatedand and hippocampi were isolated and used for further tests.

### Cell culture and microwave exposure

PC12 cells, provided by China Infrastructure of Cell Line Resource, were cultured in RPMI 1640 basic medium (Gibco, Waltham, MA) supplemented with 10% horse serum (HS; Gibco) and 5% fetal bovine serum (FBS; Gibco). To induce the formation of neuron-like cells, PC12 cells were maintained in RPMI 1640 basic medium supplemented with 10 ng/ml nerve growth factor (NGF; Sigma, St. Louis, MO) and 1% HS for 5 days. Subsequently, the neuron-like cells with neuronal phenotype (an extension of neurites) were observed.

The neuron-like cells were cultured in a six-well plate containing 2 ml medium, and randomly divided into the MW and SH groups. The cells in the MW group were exposed to 2856 MHz microwave radiation with an average power density of 30 mW/cm² for single 15 min. The SAR was calculated to be approximately 19.0 W/kg. Similar processing was conducted on cells in the SH group but without microwave radiation. Thereafter, the cells were harvested for analysis at specific time points.

### Transmission electron microscopy

At 7 days, 14 days and 1 month, rat hippocampi were isolated and 1 mm^3^ tissue blocks were collected. At 6 h, neuron-like cells cultured on removable 96-well plate were harvested. Then, the samples were successively fixed in 2.5% glutaraldehyde and 1% osmium acid, processed with graded ethyl alcohols and embedded in EPON618. After being cut into ultrathin (70-nm) sections, the samples were subsequently stained with uranyl acetate and lead citrate. The ultrastructure of rat hippocampal neurons and neuron-like cells, especially autophagosomes and autolysosomes, was observed under transmission electron microscopy (TEM) (Hitachi, Japan).

### Western blots

Total proteins were extracted from rat hippocampus at 7 days, 14 days and 1 month after exposure from neuron-like cells at 6, 12 and 24 h after exposure. Beclin1, microtubule-associated protein light chain 3 (MAP/LC3 or LC3), autophagy-related gene (Atg) 5 (Atg5), Atg7, Atg9, AMPKα2, p-AMPKα (Thr^172^), AMPKα and glyceraldehyde-3-phosphate dehydrogenase (GAPDH) were labeled with a rabbit anti-Beclin1 antibody (1:1000 dilution; #3,495; Cell Signaling Technology, CST), rabbit anti-LC3A/B antibody (1:1000 dilution; #12,741; CST), rabbit anti-Atg5 antibody (1:1000 dilution; #12,994; CST), rabbit anti-Atg7 antibody (1:1,00 dilution; #8,558; CST), rabbit anti-Atg9A antibody (1:1000 dilution; #13,509; CST), rabbit anti-AMPKα2 antibody (1:1000 dilution; #3,760; Abcam), rabbit anti-phospho-AMPKα (Thr^172^) antibody (1:1000 dilution; #2,535; CST), rabbit anti-AMPKα antibody (1:1000 dilution; #5,832; CST), and mouse anti-GAPDH antibody (1:5000 dilution; #8,245; Abcam), respectively. After incubating with the corresponding goat anti-rabbit IgG-horseradish peroxidase (HRP) (1:5000 dilution; #2,004; Santa Cruz) and goat anti-mouse IgG-HRP (1:5000 dilution; #2,005; Santa Cruz), the protein bands were recorded using an X-OMAT BT Film imaging system (Carestream, Rochester, NY). Image-Pro Plus 6.0 (Media Cybernetics, Rockville, MD) was used to analyze the integrated optical density (IOD) of protein bands.

### Autophagic flux analysis after microwave exposure

To analyze autophagic flux in neuron-like cells after microwave exposure, 50 μM chloroquine (CQ, #142116, Abcam) was added 1 h before microwave radiation to inhibit the lysosome-mediated degradation of autophagosomes. LC3-II and LC3-I expression in cells pretreated with or without CQ was detected by Western blot at 6, 12 and 24 h after microwave exposure, using rabbit anti-LC3A/B antibody (1:1000 dilution; #12,741; CST), and the ratio of LC3-II to GAPDH was calculated and statistically analyzed.

The expression and localization of LC3 protein in neuron-like cells treated with or without CQ was detected by immunofluorescence (IF) staining. Briefly, neuron-like cells grown on coverslips were harvested at 6 h after exposure and fixed with a mixture of methanol and acetone (1:1) for 10 min at room temperature. Then, the cells were labeled with rabbit anti-LC3A/B antibody (1:100 dilution; #12,741; CST) overnight at 4°C. After being washed, the cells were incubated with fluorescein isothiocyante (FITC)-conjugated goat anti-rabbit IgG secondary antibody (1:200 dilution; #0311; ZSGB-BIO; Beijing, China) for 1 h at room temperature. The cell nuclei were stained with 4′,6-diamidino-2-phenylindole (DAPI) for 5 min. Finally, the expression and localization of LC3 in cells was observed using a fluorescence microscope (DM6000, Leica, Wetzlar, Germany).

### MiR-30a expression in rat hippocampi and neuron-like cells

At appointed time points after microwave radiation, rat hippocampi were collected and neuron-like cells were harvested. Total RNA was isolated from rat hippocampi and neuron-like cells using an mirVana miRNA Isolation kit (Ambion, Waltham, MA), according to the manufacturer’s instructions. Thereafter, complementary DNA (cDNA) was synthesized using a TaqMan MicroRNA Reverse Transcription kit (Applied Biosystems, ABI, Waltham, MA). The expression of miR-30a was quantified by real-time reverse-transcript polymerase chain reaction (RT-PCR) using TaqMan Universal Master Mix II (with UNG) and TaqMan MicroRNA assays (ABI). Finally, the expression of miR-30a was normalized to that of U6.

### *In situ* hybridization

At 7 and 14 days after microwave radiation, rat brains were collected and fixed in 10% buffered formalin solution, embedded in paraffin and cut into 3-μm-thick sections. Then, miR-30a expression was assessed by *in situ* hybridization (ISH). Briefly, the sections were hybridized with miR-30a, U6, or scramble miRNA probes (Exiqon, Vedbaek, Denmark). After being labeled with anti-DIG (Boster Biological Technology, China) and stained with diaminobenzidine (DAB) (ZSGB-Bio), the expression of miR-30a and U6 was assessed in a blind manner under a light microscope (Leica). To quantify miR-30a expression, Image-Pro Plus 6.0 was used to analyze the IOD, and the expression of miR-30a was normalized to that of U6.

### Bioinformatics analysis of downstream target genes of miR-30a

To predict the target genes of miR-30a, miRanda (Version 1.9, http://cbio.mskcc.org/miRNA2003/miranda.html), miRDB (Version 5.0, http://mirdb.org/index.html), TargetScan (Version 7.1, http://www.targetscan.org/mmu_71/), and miWalk (Version 2.0, http://zmf.umm.uni-heidelberg.de/apps/zmf/mirwalk2/) analysis was conducted, and only the genes co-predicted at least by three databases were selected as candidates for further analysis. Gene naming was standardized using NCBI Entrez Gene ID. The functional enrichment analysis of the target genes of miR-30a was conducted using clusterProfiler [24] with the Kyoto Encyclopedia of Genes and Genomes.db (KEGG.db; Version 83.1, http://www.genome.jp/kegg/) [25]. Significance was assessed using hypergeometric tests and Benjamini–Hochberg correction, with a significance threshold of *P*<0.05.

### Luciferase reporter vectors and luciferase assay

HEK-293T cells were transiently transfected with pmirGLO-rPrkaa2 3′-untranslated region (3′UTR) wildtype (WT, 5′-CCUUCUGUUUACUUUUAGAA-3′), pmirGLO-rPrkaa2 3′UTR mutant type (MUT, 5′-CCUUCCAAUAGAUUUUAGAA-3′) and empty pmirGLO vectors (GenePharma, Shanghai, China) in combination with miR-30a mimics/negative control miRNAs (Ambion), using Lipofectamine 2000 (Invitrogen). Cells were lysed by Passive Lysis Buffer (Promega, Madison, WI) for 15 min. Then, firefly and *Renilla* luciferase activities were detected using a Dual Luciferase Assay kit (Promega) on a luminometer (Thermo Fisher Scientific).

### RNA transfection

At 24 h before microwave exposure, neuron-like cells were transiently transfected with 12.5 nM miR-30a mimics and negative control miRNAs (Ambion) using Lipofectamine RNAiMAX (Invitrogen). Neuron-like cells were harvested at 6 h after microwave exposure. The expression of miR-30a was analyzed by real-time RT-PCR. The protein levels of AMPKα2, p-AMPKα (Thr^172^), AMPKα, Beclin1, LC3-I, LC3-II and GAPDH were detected by Western blots.

### Statistical analysis

Data are presented as the mean ± standard error of the mean (SEM). All experiments were performed with a minimum of three independent replicates. The statistical analysis was achieved by the SPSS software (IBM, Armonk, NY, U.S.A.). For the data from two groups SH and MW with different time points, one-way analysis of variance (ANOVA) with repeated measures was used to analyze the time course, then Student’s *t* test was used to compare the differences between groups. For experiments of two-factor factorial design, two-way ANOVA was performed to analyze the effects of two factors. Besides, one-way ANOVA followed by Bonferroni’s post-hoc tests were performed to compare multiple groups. The differences were considered significant at the level of a two-sided *P*<0.05.

## Results

### Microwave radiation promoted the occurrence of autophagy in rat hippocampal neurons and PC12 cell-derived neuron-like cells

**Figure 1:**
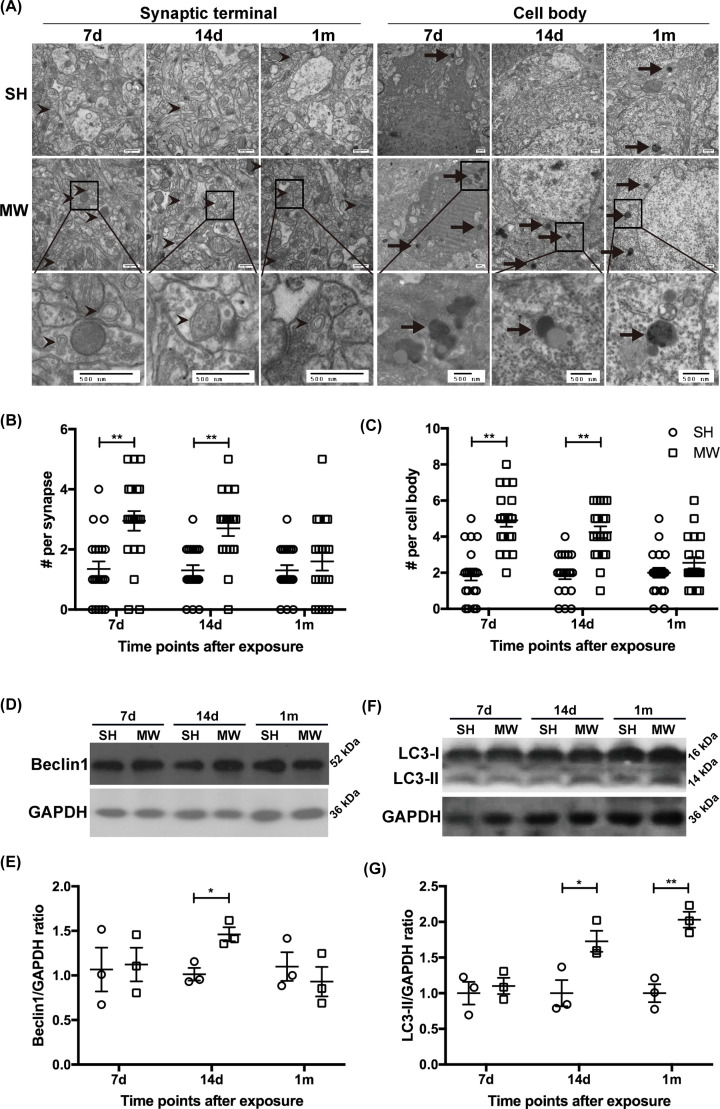
Microwaves promoted autophagy occurrence in rat hippocampal neurons (**A**) Representative images showing autophagosomes and autolysosomes in hippocampal neurons observed by TEM (scale bar = 500 nm), 7 days, 14 days and 1 month postradiation. The autophagosomes are indicated by arrows without tails, while the autolysosomes are indicated by arrows with tails. (**B**,**C**) The quantification results of the total number of autophagosomes and autolysosomes in synapse and cell body and statistical analysis. (**D**,**E**) Representative protein bands of Beclin1 and GAPDH in rat hippocampus and quantitative analysis result, on 7 days, 14 days and 1 month postradiation. (**F**,**G**) Representative protein bands of LC3-I, LC3-II and GAPDH in rat hippocampus and quantitative analysis result, on 7 days, 14 days and 1 month postradiation. The data are presented as mean ± SEM. One-way ANOVA with repeated measures was used to analyze the time course, and Student’s *t* test was used to compare the difference between the two groups SH and MW (B,C,E,G). *, *P*<0.05; **, *P*<0.01 vs the control.

First, the ultrastructure of rat hippocampal neurons was observed by TEM at 7 days, 14 days and 1 month after 30 mW/cm^2^ microwave radiation. The autophagosomes can be identified by its contents (morphologically intact cytoplasm), and the limiting membrane that is partially visible as two bilayers separated by a narrow electron-lucent cleft. The autolysosomes can be identified by its contents, partially degraded, electron-dense rough endoplasmic reticulum [26]. The number of autophagosomes and autolysosomes in neurons notably increased from 7 days to 1 month after microwave exposure. Double-membrane autophagosomes, encapsulating mitochondria, synaptic vesicles and other cytosolic constituents, were primarily distributed at the synaptic terminal of hippocampal neurons. Monolayer autolysosomes, shown as black granular or amorphous aggregates, degraded cytoplasmic components at different stages and were mainly located in cell bodies of hippocampal neurons (Figure 1A–C). To analyze the dynamic regulation of microwave radiation on autophagy, Beclin1 and LC3, two widely used molecular markers of autophagy, were analyzed in rat hippocampi. The expression of Beclin1 was up-regulated 14 days after microwave radiation, suggesting the activation of autophagy, but down-regulated at 1 month which might be attributed to a regulatory adjustment (Figure 1D,E). The expression of LC3-II significantly increased 14 days and 1 month after microwave exposure, indicating an increased number of autophagosomes (Figure 1F,G). These results suggested that 30 mW/cm^2^ microwave radiation promoted the occurrence of autophagy in rat hippocampal neurons.

**Figure 2:**
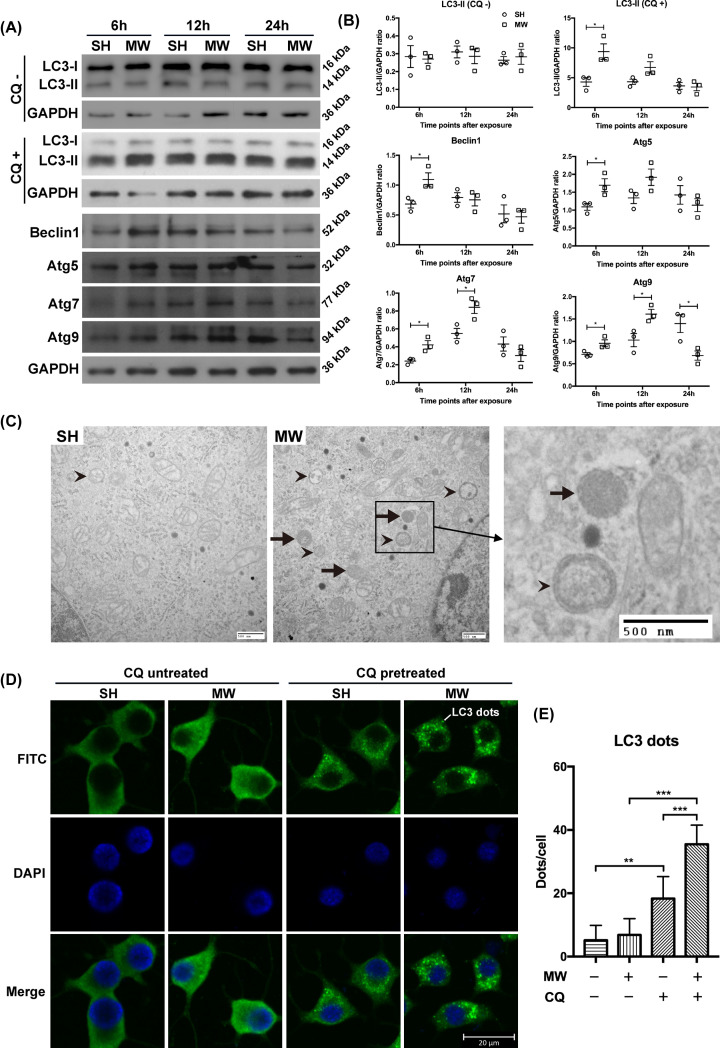
Microwave radiation activated functional autophagy in PC12 cell-derived neuron-like cells (**A**) Representative protein bands of LC3-1, LC3-II, Beclin1, Atg5, Atg7, Atg9 and GAPDH in PC12 cell-derived neuron-like cells induced by NGF, at 6, 12 and 24 h after 30 mW/cm^2^ microwave exposure. To assess ‘autophagic flux’, neuron-like cells were pretreated with chloroquine (CQ+) or equal amount of water (CQ−) 1 h before microwave exposure, and the expression of LC3-1 and LC3-II was detected by Western blots. (**B**) The statistical analysis results of protein bands in (A). (**C**) Representative images showing autophagosomes and autolysosomes in neuron-like cells at 6 h after exposure, observed by TEM (scale bar = 500 nm). (**D**) Representative images of LC3 dots in neuron-like cells at 6 h after radiation, detected by IF (scale bar = 20 μm). ‘CQ pretreated’ indicated that the cells were treated with CQ 1 h before microwave radiation, while ‘CQ untreated’ meant the cells were treated with equal amount of water. (**E**) The statistical analysis results of LC3 dots in (D). The data are presented as mean ± SEM. One-way ANOVA with repeated measures was used to analyze the time course, and Student’s *t* test was used to compare the difference between the two groups SH and MW (B). Two-way ANOVA was performed to analyze the data of two-factor factorial design (E). *, *P*<0.05; **, *P*<0.01; ***, *P*<0.001 vs the control.

The changes of autophagy in PC12 cell-derived neuron-like cells after microwave exposure was also studied. To evaluate ‘autophagic flux’, the LC3-II levels was detected in neuron-like cells pretreated with or without CQ, a lysosomal inhibitor. In the CQ-untreated cells, the LC3-II content showed no difference between the SH and MW groups. When the degradation of autophagosomes by lysosomes was inhibited by CQ, the expression of LC3-II in cells from the MW group obviously increased at 6 h but restored to basal levels at 12 and 24 h after exposure, indicating the enhanced ‘autophagic flux’ in neuron-like cells induced by microwave radiation (Figure 2A,B). The expression of autophagy markers Beclin1 was up-regulated at 6 h and restored at 12 h postradiation, and Atg5, Atg7 and Atg9 were up-regulated at 6 and 12 h after microwave exposure, indicating the enhanced expression of autophagy-related genes induced by microwaves (Figure 2A,B). Furthermore, we observed that autophagosomes and autolysosomes in neuron-like cells showed an increasing trend at 6 h after microwave exposure (Figure 2C). Through IF staining, an increase in the number of LC3 puncta (autophagosomes) was observed in neuron-like cells pretreated with CQ at 6 h after microwave radiation (Figure 2D,E). Our data suggested that 30 mW/cm^2^ microwave radiation activated functional autophagy in neuron-like cells.

### Microwave radiation inhibited miR-30a expression in rat hippocampi and neuron-like cells

**Figure 3:**
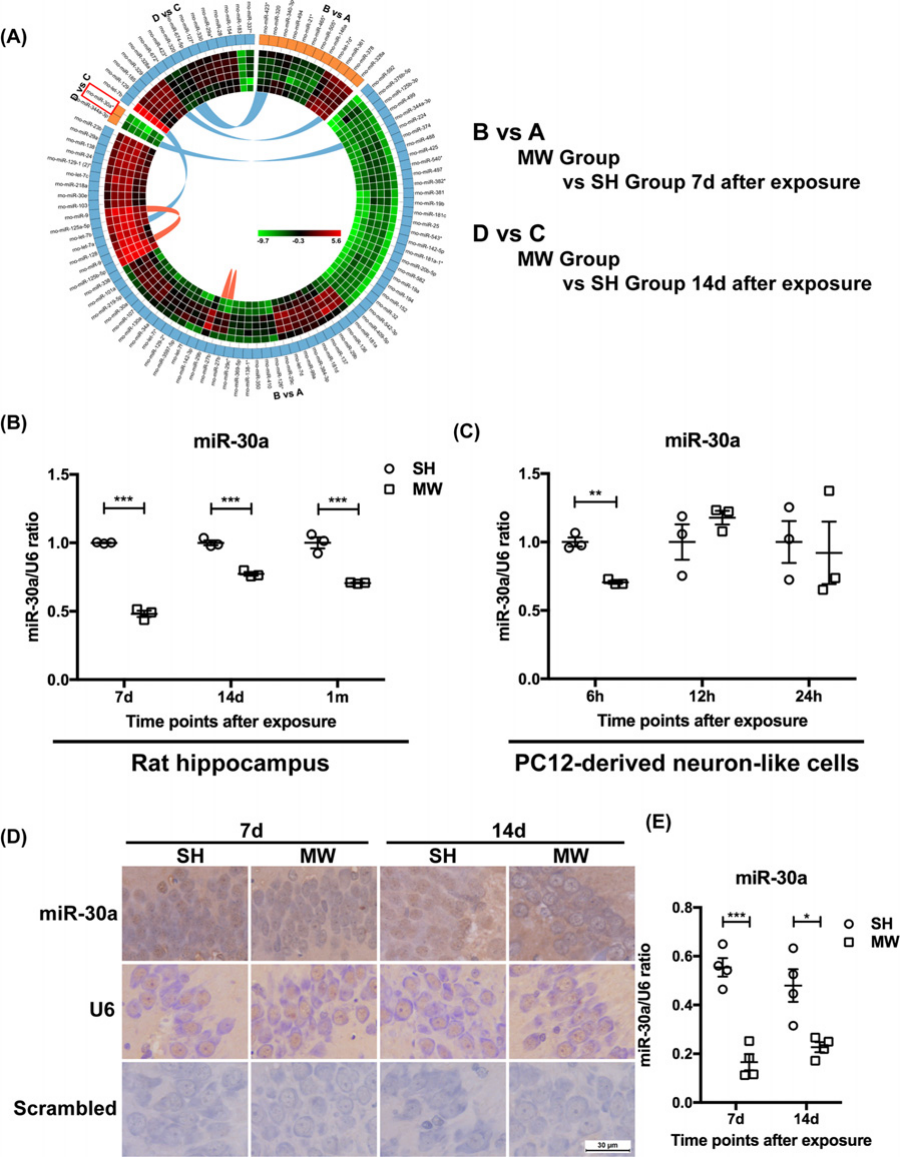
Microwave radiation inhibited miR-30a expression both in rat hippocampi and neuron-like cells (**A**) Differentially expressed miRNAs in the rat hippocampi screened by miRNA chip. (**B**,**C**) The expression of miR-30a and U6 in rat hippocampi 7 days, 14 days and 1 month after radiation and PC12 cell-derived neuron-like cells 6, 12 and 24 h after radiation, respectively, which were detected by real-time RT-PCR. (**D**) Representative images of miR-30a and U6 in rat hippocampi 7 and 14 days after radiation, detected by ISH (scale bar = 30 μm). (**E**) The quantitative analysis result of the ratio of miR-30a to U6 presented in (D). The data are presented as mean ± SEM. One-way ANOVA with repeated measures was used to analyze the time course, and Student’s *t* test was used to compare the difference between the two groups: SH and MW (B,C,E). *, *P*<0.05; **, *P*<0.01; ***, *P*<0.001 vs the control.

It has been widely reported that miRNAs play important roles in physiological and pathological events in the central nervous system [27–29]. We previously found that microwave radiation could induce cognitive dysfunction and neuron injury in rats [12]. In another study, we screened the differentially expressed miRNAs in the rat hippocampus after microwave radiation, among which miR-30a attracted our interests due to its potential regulatory roles on autophagy (Figure 3A) [14]. Based on these results, in the present study, we confirmed the changes in miR-30a levels induced by microwave radiation both *in vivo* and *in vitro*. The results suggested that microwave radiation inhibited the expression of miR-30a both in rat hippocampi and neuron-like cells (Figure 3B,C). The decreased expression of miR-30a in rat hippocampi was subsequently confirmed by ISH (Figure 3D,E). These results indicated that microwave radiation inhibited miR-30a both *in vivo* and *in vitro*, which might be the important mechanisms underlying microwave-induced autophagy in neurons.

### AMPKα2 was one of the downstream genes of miR-30a, microwave radiation increased AMPKα2 expression and AMPK (Thr^172^) phosphorylation

**Figure 4:**
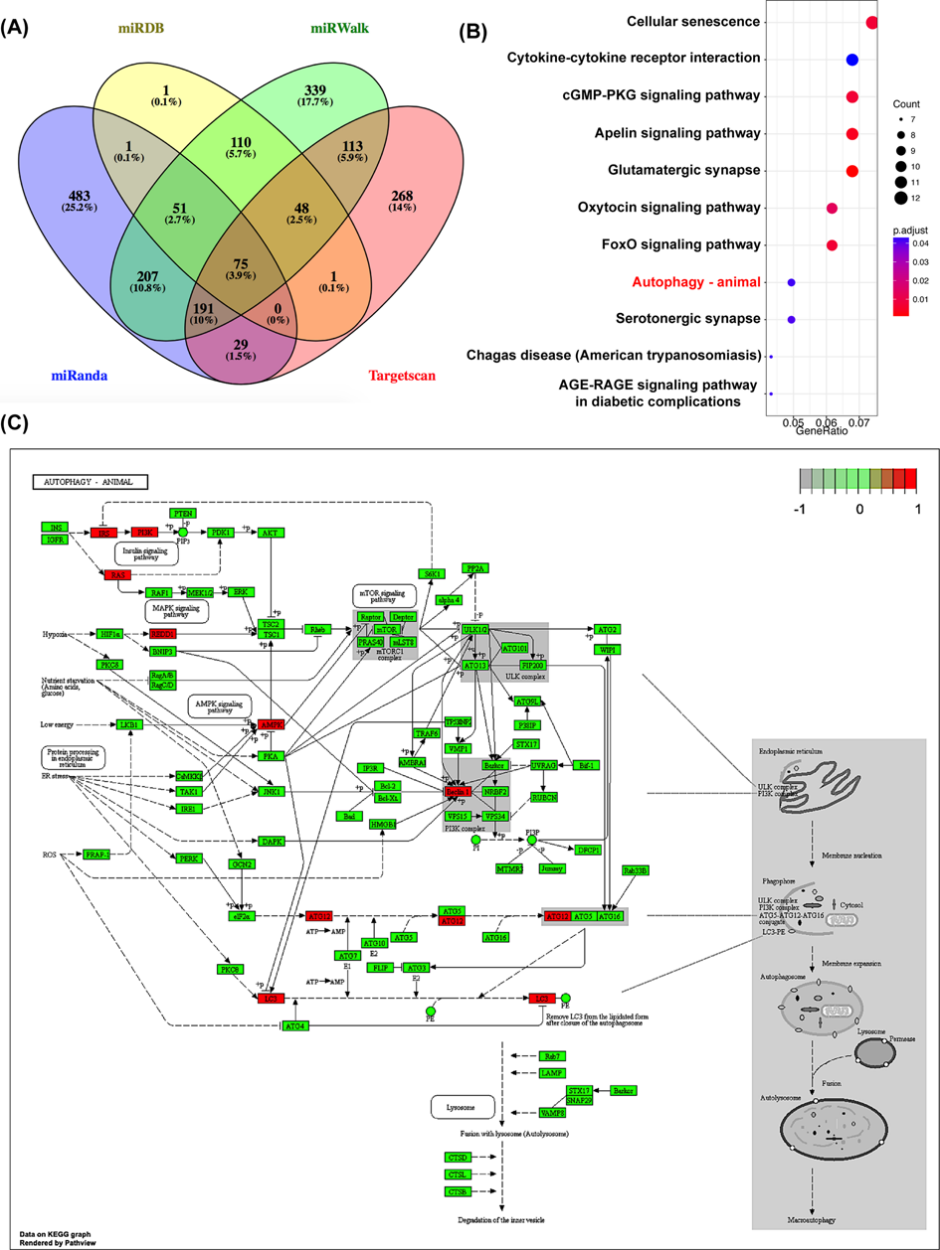
The downstream genes of miR-30a were predicted and analyzed through bioinformatics (**A**) The numbers of the predicted downstream genes of miR-30a by four databases, including miRanda, miRDB, TargetScan and miWalk. (**B**) The signal pathways regulated by miR-30a analyzed by the KEGG database. (**C**) The potential downstream genes of miR-30a involved in the regulation of autophagy, including Beclin1, Prkaa2, Irs1, Pik3r2, Rras2, Ddit4, Gabarapl2, and Atg12 are labeled in red color.

miR-30a has been shown to be an important molecule in the regulation of autophagy [15–18,30]. In the present study, we predicted the downstream genes of miR-30a using four databases, including miRanda, miRDB, TargetScan and miWalk. Subsequently, 365 target genes of miR-30a co-predicted by at least three databases were selected for KEGG analysis (Figure 4A). Our data showed that the predicted target genes of miR-30a were involved in multiple signal pathways, such as autophagy, cellular senescence, cytokine–cytokine receptor interactions, the cyclic guanosine monophosphate-dependent protein kinase (cGMP-PKG) signal pathway, and the apelin signal pathway (Figure 4B). As the potential downstream genes of miR-30a, Beclin1, Prkaa2, Irs1, Pik3r2, Rras2, Ddit4, Gabarapl2 and Atg12 appeared to play important roles in regulating autophagy. Studies have reported that Beclin1, Gabarapl2 and Atg12 mediate the induction and formation of autophagosomes. Prkaa2, encoding AMPKα2, may be involved in the regulation of autophagy through the AMPK signal pathway. REDD1, Irs1, Pik3r2, and Rras2 appears to regulate autophagy through the mammalian target of rapamycin (mTOR) signal pathway (Figure 4C) [31,32]. Among that, the role of Beclin1 in miR-30a-regulated autophagy has been widely studied [33–36]. In our research and from other scholars, microwave radiation can induce abnormal energy metabolism in neurons [20,21,37,38]. Studies have confirmed that microwave under certain conditions can promote the occurrence of autophagy, but the underlying mechanisms have yet to be clarified [12,39–42]. Accordingly, in the present study, we further explored the roles of Prkaa2 played in the miR-30a-mediated regulation of autophagy in MW neuron models, which is yet to be reported.

**Figure 5:**
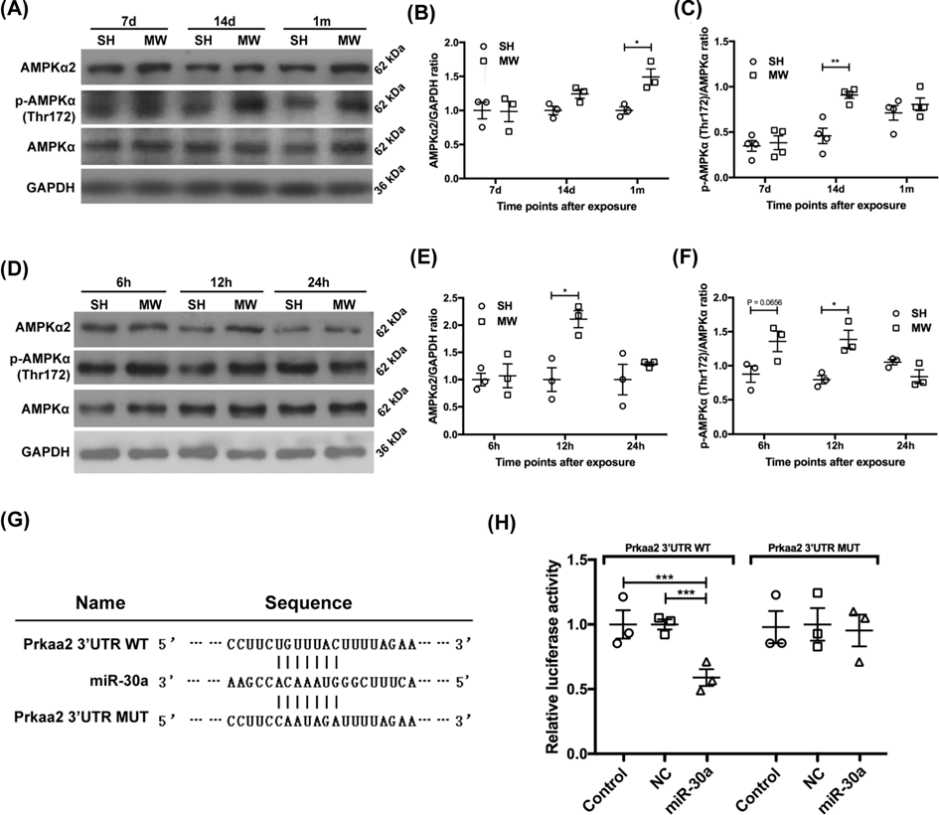
Microwave radiation increased AMPKα2 expression and AMPKα (Thr^172^) phosphorylation in rat and PC12 cell models (**A**) Representative protein bands of AMPKα2, p-AMPKα (Thr^172^), AMPKα and GAPDH in rat hippocampi on 7 days, 14 days and 1 month postradiation. (**B**,**C**) The statistical analysis results of the ratio of AMPKα2 to GAPDH and p-AMPKα (Thr^172^) to AMPKα shown in (A), respectively. (**D**) Representative protein bands of AMPKα2, p-AMPKα (Thr^172^), AMPKα and GAPDH in neuron-like cells at 6, 12 and 24 h postradiation. (**E**,**F**) The statistical analysis results of the ratio of AMPKα2 to GAPDH and p-AMPKα (Thr^172^) to AMPKα shown in (D), respectively. (**G**) The base sequences of pmirGLO-rPrkaa2 3′UTR WT, miR-30a, and pmirGLO-rPrkaa2 3′UTR MUT. (**H**) The ratio of firefly to* Renilla* luciferase activity in HEK-293T cells transiently transfected with pmirGLO-rPrkaa2 3′UTR WT, pmirGLO-rPrkaa2 3′UTR MUT and empty pmirGLO vectors in combination with miR-30a mimics or corresponding negative control miRNAs. The data are presented as mean ± SEM. One-way ANOVA with repeated measures was used to analyze the time course, and Student’s *t* test was used to compare the difference between the two groups SH and MW (B,C,E,F). One-way ANOVA followed by Bonferroni’s post-hoc tests was used to compare multiple groups in (H). *, *P*<0.05; **, *P*<0.01; ***, *P*<0.001 vs the control.

The expression of AMPKα2 was up-regulated in rat hippocampi from 14 days to 1 month after microwave exposure (Figure 5A–C), as well as in neuron-like cells at 12 h after microwave exposure (Figure 5D,F). AMPKα phosphorylation at Thr^172^ is essential for the activation of AMPK, the level of which can better reflect the changes in the activity of AMPK signal pathway [43]. In rat hippocampi, p-AMPKα (Thr^172^) levels increased 14 days after microwave exposure (Figure 5A–C). In neuron-like cells, microwave radiation induced the up-regulation of p-AMPKα (Thr^172^) levels at 6 and 12 h after exposure (Figure 5D–F). Considering the previously observed changes in autophagy, AMPK signal appeared to exhibit the same pattern as that observed for autophagy after microwave radiation, both *in vivo* and *in vitro*. These results suggested that AMPKα2 encoded by Prkaa2 was involved in the regulation of autophagy in neurons after microwave radiation. In addition, we observed that the overexpression of miR-30a reduced the luciferase activity of Prkaa2 in 3′UTR WT reporter-transfected cells but not in Prkaa2 3′UTR MUT reporter-transfected cells, suggesting an upstream regulatory role of miR-30a on AMPKα2 (Figure 5G,H). Accordingly, we hypothesized that the effects of microwave radiation on neuronal autophagy might be achieved through the miR-30a/Prkaa2/AMPKα2 pathway.

### MiR-30a overexpression inhibited AMPKα2 up-regulation, AMPKα (Thr^172^) phosphorylation, and autophagy activation mediated by microwave radiation in cell model

**Figure 6:**
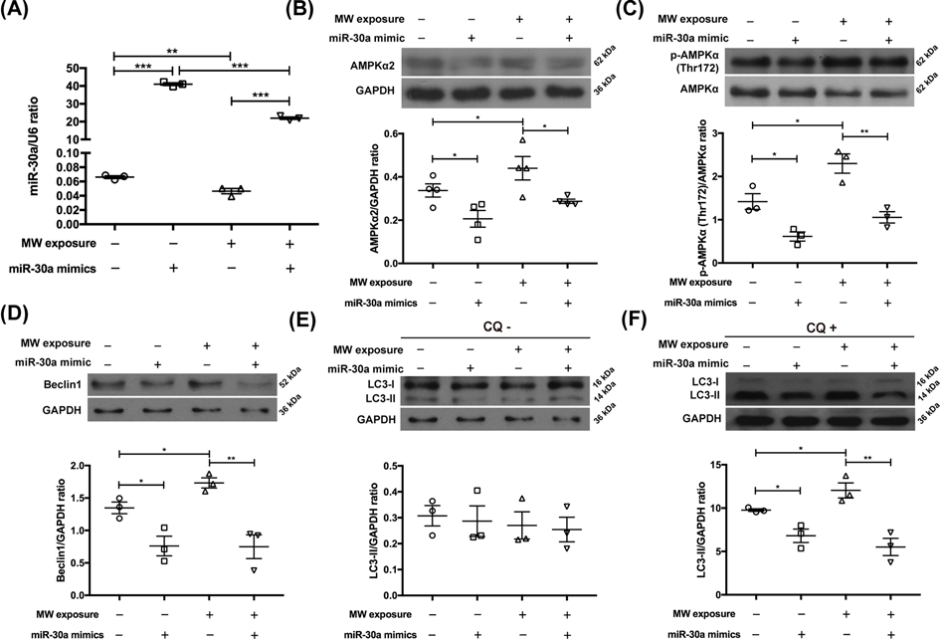
Transfection of miR-30a mimics increased miR-30a levels, reduced AMPKα2 expression, suppressed AMPKα (Thr^172^) phosphorylation and inhibited autophagy in neuron-like cells induced by microwaves (**A**) The expression of miR-30a in PC12 cell-derived neuron-like cells measured by RT-PCR. (**B**–**F**) Representative protein bands of AMPKα2, p-AMPKα (Thr^172^), AMPKα, Beclin1, LC3-I, LC3-II and corresponding GAPDHs, and related statistical analysis results. The cells were transfected with miR-30a mimics or related negative control miRNAs 24 h before 30 mW/cm^2^ microwave radiation, and harvested 6 h after exposure. CQ+ indicated that the neuron-like cells were pretreated with CQ 1 h before microwave exposure, while CQ− indicated that equal amount of water was added. The data are presented as mean ± SEM. Two-way ANOVA was performed to analyze the data of two-factor factorial design (A–F). *, *P*<0.05; **, *P*<0.01; ***, *P*<0.001 vs the control. The data in panels (D,E) are from the same sample, so they share the same GAPDH.

To investigate the underlying mechanisms that microwave-induced autophagy enhancement in neurons, we transfected miR-30a mimics into neuron-like cells 24 h before microwave exposure. It was found that miR-30a mimics significantly increased the expression level of miR-30a in neuron-like cells, suggesting that the intervention measures were effective (Figure 6A).

Few studies have reported the regulatory roles of miR-30a on the AMPK signal. In our study, miR-30a overexpression mediated by mimic transfection decreased AMPKα2 expression and inhibited AMPKα (Thr^172^) phosphorylation in neuron-like cells, indicating that miR-30a negatively regulated the activity of AMPK signal. Meanwhile, the overexpressed miR-30a inhibited microwave-induced AMPK activation (Figure 6B,C). Therefore, microwave radiation could modulate the activity of AMPK signal by suppressing miR-30a.

Furthermore, we found that miR-30a overexpression reduced Beclin1 levels in neuron-like cells and inhibited microwave-induced up-regulation of Beclin1 expression (Figure 6D). Importantly, the overexpressed miR-30a suppressed the ‘autophagic flux’ in neuron-like cells, and completely abolished microwave-induced enhancement of autophagy (Figure 6E,F). Taken together, microwave-induced autophagy in neurons was abrogated by miR-30a overexpression, and the effects of microwave on autophagy were achieved by the miR-30a/AMPK pathway.

## Discussion

Autophagy has been reported to play dual roles in pathological processes of neurodegenerative diseases. At the early stage, autophagy can enhance the degradation of denatured proteins to maintain neuronal functions, whereas at the late stage, continuous activation of autophagy ultimately induces the autophagic cell death of neurons [44–48]. Many studies have demonstrated that autophagy participates in the pathological processes of electromagnetic radiation-induced damaging effects [12,39–42]. Additionally, we previously found that autophagy-mediated degradation of synaptic vesicles was a potential mechanism of synaptic plasticity injury caused by microwaves. However, the underlying mechanisms how microwaves affect autophagy activity are yet to be clarified. In the present study, we exposed rats and PC12 cell-derived neuron-like cells to 2856 MHz microwaves with an average power density of 30 mW/cm^2^. The SAR value of the microwave radiation on rats was calculated to be approximately 10.5 W/kg, and the SAR value of neuron-like cells was 19.0 W/kg. We found that microwave radiation in the present study promoted autophagy in both rat hippocampal neurons and neuron-like cells. It seemed that autophagy occurred earlier in neuron-like cells with a larger SAR than in rat hippocampal neurons. The results of our previous study showed that hippocampal neurons had the ability to initiate self-recovery after 30 mW/cm^2^ microwave exposure [21]. Accordingly, we speculated that neuronal autophagy was most likely a protective response to microwave radiation in this study. At higher exposure dose levels, microwave radiation might overactivate autophagy, resulting in an unbalanced cellular homeostasis and ultimately leading to the irreversible injury of neurons, although further study was noted to be required [49–51].

Numerous studies have demonstrated that miR-30a negatively regulates autophagy, a process that is closely associated with the pathophysiological processes of multiple diseases, such as cerebral ischemic stroke, cancer and hepatic fibrosis [33–36]. However, the roles played by miR-30a in microwave-induced neuron injury and autophagy activation are yet to be explored. In the present study, we observed that 30 mW/cm^2^ microwave radiation inhibited the expression of miR-30a both *in vivo* and *in vitro*. Based on these results, we speculated that miR-30a might participate in the regulation of microwave-induced autophagy in neurons. The results of the present study showed that miR-30a levels were significantly down-regulated in rat hippocampal neurons from 7 days to 1 month after microwave radiation, at which time autophagy was notably activated. In addition, similar results were obtained for neuron-like cells. Furthermore, miR-30a overexpression completely abolished microwave-induced autophagy in neuron-like cells. Taken together, these results suggested that reduced miR-30 levels were an important underlying mechanism involved in the activation of autophagy mediated by microwaves in neurons.

As an important molecular target that regulates autophagy, the role of miR-30a in Beclin1-mediated autophagy has been widely studied and reported for a variety of diseases [33–36]. The results of the present study also support that decreased miR-30a levels positively regulates autophagy by promoting Beclin1 expression in neurons exposed to microwaves. However, the potential downstream target genes of miR-30a involved in regulating autophagy have yet to be systematically studied. Through bioinformatics analysis, we predicted the target genes of miR-30a that might be involved in autophagy regulation, including Beclin1, Prkaa2, Irs1, Pik3r2, Rras2, Ddit4, Gabarapl2 and Atg12. Studies have demonstrated that microwave radiation leads to abnormal energy metabolism in neurons [20,21,37,38]. In the present study, we showed that microwave radiation activated AMPK signal, a cellular energy sensor, both in rat hippocampi and neuron-like cells. There is strong consensus that AMPK signal mediates the activation of autophagy in various cells [52]. We confirmed that Prkaa2, encoding AMPKα2, was a downstream gene of miR-30a, which was verified by double luciferase reporter assay results. Moreover, microwave radiation increased AMPKα2 expression and phosphorylation (Thr^172^) both *in vivo* and *in vitro*. And miR-30a overexpression inhibited the microwave-induced activation of AMPKα2 and p-AMPKα, and abolished microwave-induced autophagy in neuron-like cells. Therefore, with the exception of Beclin1, we found that the reduced miR-30a levels after microwave exposure promoted the occurrence of autophagy by activating the AMPK signal, which had not been previously reported. Overall, in MW animal and cell model used in the study, the downregulation of miR-30a induced by microwaves increased the expression of AMPKα2 and the activation of AMPK signaling, which promoted the occurrence of autophagy and the biogenesis of autophagosomes.

## Data Availability

The data that support the findings of the present study are available from the corresponding authors upon reasonable request.

## Abbreviations

3′UTR: 3′-untranslated region
AMPK: adenosine 5′-monophosphate-activated protein kinase
ANOVA: analysis of variance
Atg: autophagy-related gene
CQ: chloroquine
CST: Cell Signaling Technology
GAPDH: glyceraldehyde-3-phosphate dehydrogenase
HS: horse serum
IF: immunofluorescence
IOD: integrated optical density
KEGG: Kyoto Encyclopedia of Genes and Genomes
MAP/LC3 or LC3: microtubule-associated protein light chain 3
miRNA: microRNA
MUT: mutant type
MW: microwave-exposed
PC12 cell: rat adrenal pheochromocytoma cell
RT-PCR: reverse-transcript polymerase chain reaction
SAR: specific absorption rate
SH: sham-exposed
TEM: transmission electron microscopy
WT: wildtype
